# Efficacy of an Online Workplace Mental Health Accommodations Psychoeducational Course: A Randomized Controlled Trial

**DOI:** 10.3390/ijerph20075317

**Published:** 2023-03-29

**Authors:** Yvonne Nichole Faller, Vanessa Peynenburg, Eric Tessier, David Thiessen, Heather D. Hadjistavropoulos

**Affiliations:** 1 Department of Psychology, University of Regina, 3737 Wascana Parkway, Regina, SK S4S 0A2, Canada; vanessa.peynenburg@uregina.ca (V.P.); tessiere@uregina.ca (E.T.); heather.hadjistavropoulos@uregina.ca (H.D.H.); 2 Department of Mathematics & Statistics, University of Regina, Regina, SK S4S 0A2, Canada; thiesdav@uregina.ca

**Keywords:** workplace mental health, workplace accommodations, online psychoeducation

## Abstract

Workplace accommodations can improve work functioning for employees with mental health concerns, yet few employees receive accommodations. The current study examined the benefits of providing education on workplace accommodations. In total, 89 participants with symptoms of depression and/or anxiety were randomized to an online psychoeducation course or wait-list control (WLC). The course provided education on symptoms, accommodations, tips for requesting accommodations and making disclosures, and coping strategies. Primary outcomes included the impact of the course on requesting and receiving accommodations, accommodation knowledge, self-stigma, and workplace relationships at 8 weeks post-randomization. Additional analyses examined the impact of the course on symptoms, absenteeism, presenteeism, and self-efficacy and whether supervisory leadership and organizational inclusivity impact disclosure and accommodation use. Participants in the course reported improvements in accommodation knowledge, self-efficacy, and presenteeism compared to the WLC. Both groups reported reduced self-stigma and increased disclosures over time. Specifically, partial disclosures were associated with supportive organizations and supervisors. No group differences were found on accommodation use, symptoms, workplace relationships, or comfort with disclosure. Few participants made accommodation requests, therefore a statistical analysis on requesting or receiving accommodations was not performed. Overall, providing psychoeducation has the potential to assist individuals with depression and anxiety who may require workplace accommodations, but further research is required.

## 1. Introduction

Existing research on workplace accommodations has focused on accommodations for physical disabilities with limited research on mental health accommodations [1,2]. In terms of the research on mental health accommodations, the focus has been on individuals struggling with serious mental health conditions (e.g., schizophrenia) who require the assistance of a job coach in acquiring and retaining work within large organizations [3,4]. Additionally, most studies have only documented the types of accommodations provided and frequency of use, with limited discussion of how the accommodation has impacted employee well-being [2,3,5]. Missing from the literature is information on how other more common mental health disorders (e.g., depression and anxiety) are accommodated, how to improve accommodation usage, and the impact of receiving an accommodation on employee work functioning, symptomology, and well-being.

Mood and anxiety disorders are highly prevalent psychological disorders, with a reported yearly prevalence of up to 11.3% in Canada [6]. There are many personal and/or work biopsychosocial factors (e.g., stress) that may contribute to mood and anxiety disorders [7,8]. Functional impairments due to depressive and anxiety symptoms have a significant and harmful impact on an individual’s occupational, social, and personal well-being [2,9,10]. For instance, a recent study on the correlates of quality of life in anxiety disorders reported that individuals with anxiety disorders experience more distress and have more difficulty regulating their emotions, which negatively impacts their overall functioning [11]. Moreover, employees with anxiety and/or depression symptoms are more likely to engage in presenteeism, resulting in lost productivity and increased costs to the employer [10,12,13].

Workplace accommodations may be one way to mitigate the impact of anxiety and mood disorders [14]. Workplace accommodations include any reasonable accommodations (e.g., flexible work schedules, job sharing) or modifications to a work environment (e.g., quiet spaces, workspace free of distraction) that diminish barriers to employment, and that enable and support individuals with a mental health condition to work more effectively while not causing undue hardship for the employer [5,15]. In one Canadian study, the most frequently reported accommodations for employees with depressive symptoms included flexible work schedules, modified tasks, and changes to job requirements and work environments [16].

Importantly, there is evidence that the implementation of workplace accommodations can lead to improved mental health symptoms, job tenure and interpersonal relationships [2,4]. For instance, Chow, Cichocki [17] found that people with job accommodations for a mental health disorder worked 7.68 more hours each month, their job tenure was 31% longer, and their risk of job termination was reduced by nearly 13% compared to those with no accommodations. In another study, it was found that workplace accommodations were associated with increased job satisfaction, quality of work life, organizational culture, improved relationships at work, and enhanced organizational reputation [18,19]. Specifically, Schartz, Hendricks [19] reported that 69.3% of respondents reported improved interpersonal relationships with coworkers, while 60.7% reported increased company morale after implementation of physical and mental health accommodations. A subsequent study by Solovieva, Dowler [15] corroborated these results. Additionally, compared to an unhealthy workplace, which is associated with negative financial impacts [10], most accommodations are low-cost or free to implement and are often associated with economic benefits or rewards [3,20]. Despite the benefits of accommodations, many employees may be unaware of accommodations they can access [14,21] or may choose to remain silent about their mental health concerns due to fear of stigma [22,23].

In contrast, disclosing a mental health concern may help to facilitate the accommodation process and is often a precursor to receiving many workplace accommodations [5,24,25]. For instance, in one study examining the predictors for receiving job accommodations, disclosure was the strongest predictor and increased the probability of receiving an accommodation by 24.5% [5]. In a systematic review of 48 empirical research articles, Brohan, Henderson [26] identified several predictors for disclosure. Specifically, they found that workers were more likely to disclose in supportive work environments, particularly if their performance was impacted by their mental health symptoms (i.e., symptoms were more severe). To help facilitate disclosure, and tangentially accommodations, information regarding workplace mental health disclosures and recommendations from MacDonald-Wilson, Russinova [27] were included within the psychoeducational course content.

The current randomized controlled trial explored the efficacy of an online psychoeducational course on workplace mental health accommodations, called the “Workplace Coping Strategies Course” (WCSC), which aimed to improve employees’ knowledge and use of mental health accommodations for anxiety and depression symptoms relative to a wait-list control (WLC) group. The primary hypotheses were that participants in the WCSC relative to WLC would: (1) report making more accommodation requests and would receive more accommodations; (2) have lower self-stigma attitudes; (3) have increased knowledge of accommodations for anxiety and/or depression; and (4) have decreased incidences of interpersonal conflict at work at an 8-week follow-up. Second, it was hypothesized that if employees were provided with accommodations to address their mental health concerns, they would experience improvements in presenteeism, self-efficacy, and anxiety/depression symptoms. Finally, it was hypothesized that higher ratings of supportive supervisory leadership and organizational inclusivity would be associated with higher rates of receiving accommodations and workplace disclosures.

## 2. Materials and Method

### 2.1. Research Design and Ethics

The study used a randomized controlled trial design with an 8-week follow-up to examine the efficacy of the WCSC relative to WLC for Canadian employees who self-reported anxiety and/or depression symptoms. An 8-week timeline was chosen as it was believed this was an adequate amount of time to request accommodations and for improvements to be assessed. The course was offered via the Online Therapy Unit at the University of Regina which delivers various internet-delivered mental health treatment programs. The present study, which ran from October 2019 until September 2020, proceeded after institutional ethics approval and trial registration (Clinical Trials.gov ID: NCT04122482).

### 2.2. Participants

#### 2.2.1. Recruitment

Participants (*n* = 89) were recruited through social media, traditional media, speaking engagements, email campaigns, paid advertisements, public and private sector organizations, and organic traffic to the unit website. Recruitment material included information about the WCSC, eligibility criteria, potential benefits of the program, and a link to the website where prospective participants could register for the WCSC (www.onlinetherapyuser.ca).

#### 2.2.2. Eligibility

Screening/eligibility measures were administered via REDCap, a secure platform for managing online surveys, to determine eligibility. The online screening questionnaire began with a consent form and included basic eligibility questions. Inclusion/exclusion criteria at this stage required that the participant: (1) was a Canadian resident; (2) was at least 18 years of age; (3) had access to a computer and the internet; (4) expressed comfort using technology; (5) self-reported at least moderate symptoms of anxiety or depression; and (6) self-reported lost productivity at work. Given that workplace accommodations are legally required only for justifiable disabilities [28], eligible participants had to endorse at least moderate symptoms of anxiety and/or depression by scoring ≥10 on either the Generalized Anxiety Disorder Questionnaire 7-item (GAD-7; [29]) or the Patient Health Questionnaire 9-item (PHQ-9; [30]). Ineligible participants were provided with an explanation for their ineligibility and encouraged to contact the researcher via email if they had any questions or concerns.

Participants who met basic eligibility requirements were immediately directed to complete the remainder of the online screening, including questions about demographics, symptoms, and their clinical history. At the end of the screening, participants were asked to select a date to complete a follow-up telephone screening to further confirm inclusion and exclusion criteria and provide details of the study over the phone.

During the telephone screening, participants were excluded from the study if: (1) symptom thresholds were not met; (2) risk of suicidality was high; (3) they endorsed symptoms of mania and/or psychosis; or (4) they presented evidence of an untreated addiction. Participants who scored a 3 on item 9 of the PHQ-9 were further assessed for suicidality using the Revised Suicide Behaviours Questionnaire (SBQ-R; [31]).

#### 2.2.3. Randomization

Prior to the start of recruitment, the research team developed an assignment schedule using a computerized randomization generator (http://www.randomization.com) URL (accessed on 1 October 2019) by following the Consolidated Standards of Reporting Trials guidelines [32]. The assignment schedule was concealed from the primary researcher and uploaded to the REDCap system.

#### 2.2.4. Sample Size

**Figure 1:**
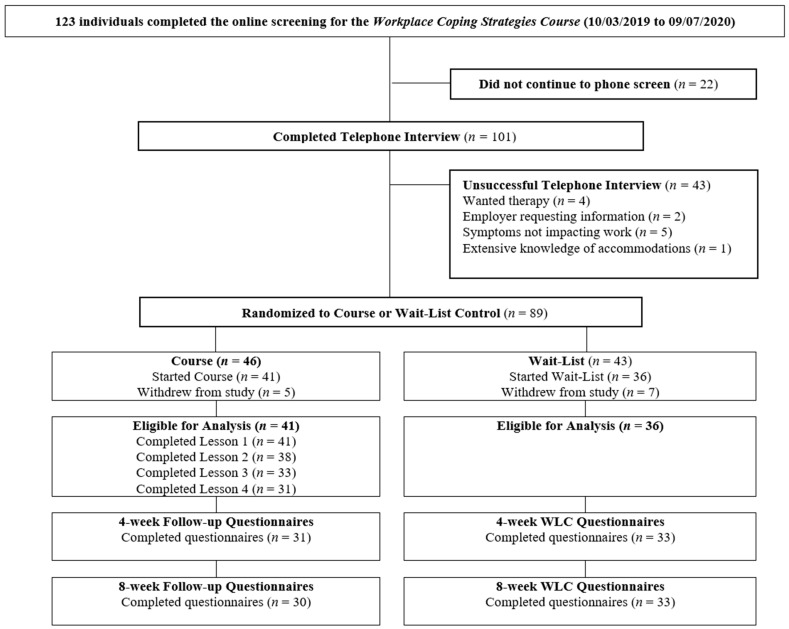
Participant flow.

Participant flow for the WCSC can be found in Figure 1. Of the 123 participants who completed the initial online screening, a total 89 participants were eligible for the study and provided consent to participate. The eligible participants were randomly assigned to either WCSC (*n* = 46) or WLC (*n* = 43). A small percentage of participants did not start the intervention (*n =* 6 from treatment group; *n =* 14 from waiting list group) after randomization. Overall, data from 41 participants in the treatment group and 36 participants in the waiting list group were eligible for analysis. Participants were not compensated.

In early March 2020, the World Health Organization declared a global pandemic. Recruitment had been ongoing for approximately 5 months prior to the pandemic and continued for an additional 6 months after the pandemic was declared. Of the 77 participants included in the analyses, 23 participants (30%) had completed the prescreen questionnaire and 5 participants (6%) had completed the study before 1 March 2020.

#### 2.2.5. Power

A power analysis was conducted using methods developed for GEEs. To calculate power for GEE, the longpower package in R was used [33,34,35,36]. The formulas for the longpower package in R require specifying the standard deviations, working correlation matrix, and differences in regression coefficients for the 2 groups. The standard deviations and correlation matrix were chosen by using data collected in the wait-list condition for the accommodation knowledge measure. Using the accommodation knowledge data, a correlation between measures of 0.6 and standard deviation of 2.6 was used. For the difference in regression coefficients, values that would result in a medium Cohen’s d between groups and the week-4 and week-8 measurements were used. Multiplying 0.5 by the estimated standard deviation of 2.6 gave a difference in regression coefficients of 1.3. Retaining an alpha of 0.05 and a power of 0.80, the total sample size required was 76. A post-hoc test of the power to detect an effect size of 0.32 with the current sample size of 77 resulted in a power of 0.7.

### 2.3. Intervention

The 4-lesson WCSC was first developed by the primary researcher using evidence-based literature (e.g., [27]) and reputable websites (e.g., Job Accommodation Network (JAN)). The content was then refined by the primary researcher using feedback on content, structure and clarity of the WCSC provided by a total of 29 legal, human resource, and management professionals as well as practicing therapists.

The WCSC included lessons aimed at: (1) identifying anxiety and depression in the workplace (symptoms, behaviours, and prevalence); (2) providing information about accommodations for anxiety and depression in the workplace (including accommodation examples, relevant legislation, benefits of accommodations, potential barriers to receiving accommodations, and strategies to overcome barriers); (3) mental health disclosure and accommodation requests (including strategies for requesting accommodations, information regarding how and when to request accommodations, pros and cons of requesting accommodations, and the level of disclosure that may be required for accommodations); and (4) symptom management (including evidence-based strategies for managing anxiety and depression, with or without accommodations). Evidence-based strategies employed in the 4th lesson included thought challenging and progressive muscle relaxation (PMR). Thought challenging or cognitive reframing is a strategy employed in cognitive behavioural therapy (CBT); a meta-analysis by Joyce, Modini [37] suggests that there is substantial evidence to support the efficacy of workplace CBT interventions in reducing symptoms of anxiety and depression. PMR has also been demonstrated to be an effective workplace intervention for anxiety and depression, as well as reducing overall workplace strain [38,39]. Lesson 4 also included information on managing work expectations, reducing procrastination, goal setting, and building work relationships, as use of these strategies may reduce the need for a formal accommodation [40]

#### Procedure

Lesson 1 of the WCSC was provided to those randomized to this group immediately after randomization. Each subsequent lesson unlocked in succession, 24 h after lesson 1 with automated emails sent to inform participants of each lesson. Participants were encouraged to complete all 4 lessons within 4 weeks of receiving the content; however, they had access to the WCSC lessons for up to 8 weeks. At 8 weeks, the control group was given access to the WCSC. All participants received questionnaires at 4 weeks and 8 weeks post-randomization. If measures were not completed, they received up to 2 reminder emails and 1 phone call to encourage completion of the measures.

### 2.4. Measures

Measures listed below were administered before randomization and at 4 weeks and 8 weeks post-randomization (Several open-ended questions were asked but are not described in this paper. Participants were asked about the nature of the accommodations they received, as well as about barriers and facilitators of receiving accommodations. They were also asked to provide feedback on the course).

#### 2.4.1. Demographics

Participant demographic information was collected during screening and included age, gender, marital status, education, ethnicity, location, employment characteristics (e.g., position, work functioning), and mental health characteristics (e.g., duration of mental health concerns).

#### 2.4.2. Accommodation Questions

Participants were asked to rate their level of knowledge of accommodations and comfort level with requesting an accommodation on separate 10-point scales. Consistent with previous research [20], participants were asked a yes/no question related to whether they had requested any change or accommodation in the workplace to better meet their mental health needs. This question was asked at baseline and then at 8 weeks. If the respondent provided a “no” response, they were prompted to answer why they had not requested this change or accommodation. If the respondent answered “yes”, they were asked if the change or accommodation was made.

#### 2.4.3. Self-Stigma of Mental Illness Scale Short Form (SSMIS-SF; [41])

The SSMIS-SF consists of 20 items resulting in 4 subscales that assess self-stigma in terms of: awareness of common stereotypes of mental health concerns (SSMIS-Aware), level of agreement with common mental health stereotypes (SSMIS-Agree), how much they apply the stereotypes to themselves or internalize them (SSMIS-Apply), and the degree to which the respondents who have internalized the stereotype are hurt by it (SSMIS-Hurt). The total score for each construct was calculated by summing respondents’ answers on a 9-point Likert-type scale. A higher score indicates higher levels of self-stigma on each specific construct. The SSMIS-SF has good internal consistency across studies that included diverse mental health disorders and participant populations [41]. In the current study, Cronbach’s α was 0.90 at baseline.

#### 2.4.4. Worker Relations Scale (WRS; [42])

The WRS consists of 9 items rated on a 1 to 7 scale assessing the relationships between the employee, their coworkers, supervisors, and the organization, with higher total scores representing more positive worker relations [42]. The WRS has demonstrated adequate psychometric properties [42]. In the current study, Cronbach’s α for the WRS scale was 0.86 at baseline.

#### 2.4.5. Work Performance

The World Health Organization and Work Performance Questionnaire (WHO HPQ) Clinical Trials Version was administered to assess absenteeism and presenteeism rates because of its focus on work impairments due to illnesses (e.g., depression) treated within clinical trials [43]. This version consists of 13 questions with cut-off scores of ≥3 for absenteeism and ≤ 40 for presenteeism [44]. Higher scores on absenteeism measures represent a higher number of days/hours lost due to disability [44]. In contrast, higher presenteeism scores represent a lower amount of lost performance. Overall, the WHO HPQ has been shown to have excellent validity, reliability, and sensitivity to change [43]. Notably, absenteeism was measured but a review of the data suggested the absenteeism data could not be used because of inconsistent responses from participants (see limitations).

#### 2.4.6. New General Self-Efficacy Scale (NGSE; [45])

The NGSE scale consists of 8 items rated on a 1 to 5 scale assessing how much respondents believe in their ability to achieve goals despite difficulties, with higher scores indicating greater self-efficacy, which is indicative of better workplace performance [45]. In a study comparing the NGSE to 2 other self-efficacy scales, Scherbaum, Cohen-Charash [46] reported that the NGSE had good internal consistency (α = 0.85), greater discrimination parameters, and the least amount of variability as compared to the other 2 measures. In the current study, Cronbach’s α was 0.90 at baseline.

#### 2.4.7. Supervisor Servant Leadership Scale (SSLS; [47])

The SSLS was used to assess the leadership qualities of organizational supervisors. It consists of 7 items that are rated on a 5-point Likert-type scale. A total score is generated from the 7 items, with higher scores indicating more supportive supervisors. Previous psychometric analyses of this measure are not available. Cronbach’s α was 0.89 at baseline.

#### 2.4.8. Climate for Inclusion Scale (CIS; [48])

The CIS assesses inclusion of diversity within organization and consists of 15 items rated on a scale from 1 (strongly disagree) to 5 (strongly agree). The items were summed to create a total score, with higher scores indicative of a more inclusive organization. Cronbach’s alpha for the integration of differences subscale at baseline in the current study (α = 0.94) was the same as has been reported previously [48].

#### 2.4.9. Disclosure

Participants were asked 1 question regarding their comfort with fully disclosing a mental health condition and 1 question about their comfort with partial disclosure (e.g., only disclosing the acceptable parts of their condition). Both questions were rated on a 4-point Likert scale (1 = very uncomfortable; 4 = very comfortable) and have been used in previous studies of mental health disclosure (e.g., [27,49,50]). Also consistent with previous research [27,50], participants were asked to indicate whether they had made no disclosure, a selective disclosure (i.e., informing others about the presence of a medical condition or the limitations/barriers/restrictions they are experiencing), or a full disclosure.

#### 2.4.10. PHQ-9

The PHQ-9 consists of 9 items rated on a 0 to 3 scale and is used to assess the severity of depression symptoms [30]. A total score ≥ 10 is indicative of clinically significant levels of depression [30,51]. The PHQ-9 has been found to have sound psychometric properties [30]. Cronbach’s α was 0.85 at baseline in the current study.

#### 2.4.11. GAD-7

To assess the severity of anxiety symptoms, the GAD-7 [29] was administered and consisted of 7 items rated on a 0 to 3 scale. A total score ≥ 10 is indicative of clinically significant levels of anxiety [29,52,53]. The GAD-7 has good overall psychometric properties [29]. In the current study, Cronbach’s α was 0.84 at baseline.

#### 2.4.12. SIAS-6 and SPS-6

The Social Interaction Anxiety Scale (SIAS-6; [54]) and Social Phobia Scale Short Form (SPS-6; [54]) were used to assess social anxiety. Each scale consists of 6 self-report items rated on a 0 to 4 scale and has good psychometric properties [54]. A score ≥ 7 on the SIAS-6 and ≥2 on the SPS-6 is indicative of social phobia [54]. Cronbach’s α was 0.90 at baseline in the current study for the 2 scales.

### 2.5. Analyses

All statistical analyses were completed using the Statistical Package for the Social Sciences (SPSS) version 26 and R version 4.1.0. Descriptive statistics were used to describe the sample in terms of background characteristics. Group differences in baseline demographic and clinical variables were assessed using independent samples t-tests for continuous (e.g., age) variables and Pearson chi-square tests for categorical (e.g., ethnicity, province of residence) variables. Next, the number of participants requesting and receiving n accommodation was examined, but no group comparisons were made given the low frequency of both requesting and receiving accommodations.

Generalized estimating equation models (GEEs) [55] were used to examine hypothesized differences in improvements between groups on knowledge of workplace accommodations, decreased self-stigma attitudes, and improved workplace relationships. GEEs were also used to examine whether the groups differed in changes in presenteeism, comfort with and rates of disclosure, self-efficacy, depression, generalized anxiety, and social anxiety. Improvements on the PHQ-9, GAD-7, WRS, WHO HPQ (absent partial day), and all SSMIS-SF subscales were modelled as proportional changes from baseline to weeks 4 and 8 using a Gamma distribution with log link function [56]. These models predict larger improvements for participants with worse baseline symptoms. For the accommodation knowledge variable, NGSE, and disclosure variables an assumption of change being proportional to baseline measures would assume that participants who had better baseline measures would show larger improvements from treatment, which contrasts with Karin, Dear [56]. Thus, for these variables, we modelled changes over time linearly using a Gaussian distribution with identity link. All GEE models used exchangeable working correlation structures and robust sandwich estimates of standard errors. For all GEE models, hypothesis tests on group differences in improvements were performed by Wald tests on Time*Group coefficients. Estimates of Cohen’s *d* effect sizes were also calculated.

A modified intention-to-treat (ITT) analysis was used to account for participant noncompliance and missing data [57], such that an analysis was undertaken of all participants who did not withdraw from the study (*n* = 5 withdrew from the course and *n* = 7 withdrew from the WLC). All participants were asked to complete questionnaires at each observation time and all completed or partially completed questionnaires were analyzed.

Logistic regression models were conducted to examine whether baseline ratings of supervisor leadership (SSLS) and organizational inclusiveness (CIS) were associated with requesting or receiving mental health accommodations. A linear regression model was used to examine whether ratings of supervisor leadership and organizational inclusiveness predicted comfort with and rates of disclosing mental health condition(s). Specifically, logistic regressions were used in models for requesting and receiving accommodations given these outcome variables are dichotomous, and linear regressions were used for comfort disclosing and rates of disclosure given these outcome variables are continuous.

## 3. Results

### 3.1. Participant Characteristics

Demographic and clinical baseline data are presented in Table 1. Participants ranged in age from 20 to 60 years, with a mean age of 43.84 years (*SD* = 10.64). Most participants identified as female (*n =* 60; 78%) and described their ethnicity as European (*n* = 59; 76.6%). Most participants also reported working full time (*n* = 51; 67%). The sample worked in diverse fields including technical/administrative support positions (*n* = 22; 28.5%), professional positions (*n* = 20; 26%), senior management (*n* = 19; 24.7%) and sales (*n* = 16; 20.8%) and for small (*n* = 46; 59%) companies. Participants reported several full (*M* = 5.61; *SD* = 9.92) or partial (*M* = 3.52; *SD* = 8.27) day absences from work and performing at almost half their ability (*M* = 56.49; *SD* = 24.64) due to mental health symptoms during the previous 28 days. Over half the total sample reported experiencing symptoms of anxiety (*n* = 58; 75.3%) and depression (*n* = 47; 61%) for more than 1 year. Participants also reported mean levels of depression (*M* = 12.77, SD = 5.53) and generalized anxiety (*M* = 10.86; SD = 4.54) at baseline that were moderate in nature. As illustrated in Table 1, there were no statistically significant baseline differences between the groups.

### 3.2. Primary Outcome Measures

Too few participants requested accommodation (*n* = 12 at baseline, *n* = 16 at week 8) to permit a statistical analysis of the impact of the WCSC on requesting or receiving an accommodation.

Table 2 presents estimated marginal means, standard deviations, and percentage changes from baseline to post-treatment. Table 3 shows Cohen’s *d* effect sizes for the outcome measures. In terms of significant findings, there were statistically significant group-by-time interactions in the GEE models for accommodation knowledge (*p* = 0.01), presenteeism (*p* = 0.04), and self-efficacy (*p* = 0.01). On these 3 measures, the participants in the WCSC reported improvements from baseline to weeks 4 and 8, while the WLC did not report statistically significant changes. These results suggest that the WCSC was effective in improving measures of accommodation knowledge, presenteeism, and self-efficacy. Between-group Cohen’s *d* effect sizes were large at week 8 on accommodation knowledge and small on presenteeism and self-efficacy.

In terms of other statistically significant findings, there was a main effect of group on the SSMIS-Aware scale (*p* < 0.001), reflecting lower SSMIS-Aware scores for the WCSC group than the WLC at baseline. No other main effects of group or interactions of group with time were significant.

#### Psychological Distress/Symptomology

The GEE models found statistically significant main effects of time on the GAD-7 (*p* < 0.001) and PHQ-9 (*p* < 0.001) but not significant time-by-group interactions on either measure (*p* > 0.42), indicating both groups improved on measures of anxiety and depression over time. There were no significant main effects or interactions on the SIAS-6 or SPS-6 (*p* > 0.21). See Table 3 for details.

### 3.3. Role of Supervisory Leadership and Organizational Inclusiveness

In the logistic regression, neither supervisory leadership nor organizational inclusiveness were significant predictors of accommodation requests (SSLS: *B* = −0.28 (*SE* = 0.26), *z* = −1.07, *p* = 0.28; *B* = −0.16 (*SE* = 0.26), *z* = −0.63, *p* = 0.54) or receiving accommodations (SSLS: *B* = −0.07 (*SE* = 0.32), *z* = −0.23, *p* = 0.82; CIS: *B* = 0.45 (*SE* = 0.34), *z* = 1.33, *p* = 0.18). The results of the linear regressions indicated that supervisory leadership (SSLS) and organizational inclusiveness (CIS) explained 13.2% of the variance in ratings of comfort with full disclosure (*R*^2^= 0.13, *F*_(3, 73) =_ 3.69, *p* = 0.02), 7.6% of the variance in ratings of comfort with partial disclosure (*R*^2^ = 0.08, *F*_(3, 73)_ = 1.99, *p* = 0.04), and 0.3% of the variance in rates of disclosure (*R*^2^= 0.003, *F*_(3, 73)_ = 0.07, *p* = 0.97).

## 4. Discussion

The current study addresses a gap in the literature by evaluating an online psychoeducational course aimed at improving knowledge and use of accommodations for symptoms of anxiety and depression. Consistent with our hypotheses, the WCSC was found to result in improvements in accommodation knowledge, presenteeism, and self-efficacy compared to the WLC. Findings related to self-efficacy appear particularly promising as self-efficacy is known to contribute to overall employee well-being and has protective properties in terms of buffering employees against workplace stressors [58]. Improvements in presenteeism also represent a beneficial contribution of the course. Presenteeism, or attending work while ill, is correlated with anxiety, depression, and job dissatisfaction [10,59] and reducing instances of presenteeism improves productivity in the workplace [60].

It is unknown what specific information provided within the WCSC course led to the benefits noted above. Therefore, it is advisable that employees and employers receive and/or provide education on anxiety and depression in the workplace (symptoms, behaviours, and prevalence); possible accommodations and the process for receiving an accommodation; and evidence-based strategies for symptom management (e.g., thought challenging, PMR, managing work expectations). By having and/or providing this information, it is possible that the recipients may also experience similar benefits.

While benefits to the WCSC were found, other benefits were not realized. Reductions in self-stigmatizing attitudes, depression, and anxiety were reported across both the WCSC and WLC. Unfortunately, it was not possible to assess whether the WCSC positively impacted requests or receipts of accommodations within the 8-week assessment period, as the number of disclosed requested accommodations was too low. This finding was surprising given past literature showing employees are more likely to access accommodations if they have increased awareness of what options are available to them [61]. Low rates of requested and received accommodations may be explained in part by the COVID-19 pandemic, with many businesses shifting to work-from-home environments, which can allow for greater flexibility for work environment, modified work tasks, and more flexible work hours without a formal accommodation [62,63,64]. It may also be the case that participants’ awareness of accommodations increased as a result of the WCSC; nevertheless, they did not identify the need for an accommodation [40] and therefore did not request one. In the current study, we failed to find main effects for improved workplace relationships over time or between groups; thus, impaired workplace relationships may have acted as a barrier to seeking accommodations. Tulk, Mantler [65], reported that knowing about a coworkers’ anxiety/depression diagnosis led to doubts about their work abilities and increased perceptions of dangerousness. As such, it may not be the accommodation itself that creates interpersonal strain at work, but rather the disorder being accommodated that increases the risk for workplace conflict. Ultimately, future versions of the WCSC should be revised to include greater attention to workplace relationships, which may lead to higher rates of accommodation requests and approvals.

While supervisory leadership and organizational inclusiveness did not significantly predict accommodation requests or receiving an accommodation, these two factors did help explain some of the variance in participants’ comfort with making a full or partial disclosure. It has been previously noted that disclosure is the strongest predictor for receiving an accommodation [5]; thus, these findings are important when considering factors that might impact successful receipt of an accommodation.

## 5. Study Limitations

There were some methodological limitations to the study that should be acknowledged. First, participants were asked if they had requested an accommodation, and if they answered ‘yes,’ they were asked if the accommodations had been received. In hindsight, this meant that employee-implemented accommodations that were not specifically requested were not measured. Second, in the post-questionnaires, instead of asking participants what accommodations were requested or received, participants were asked to identify the accommodations they were aware of. Consequently, it is unknown what accommodations were received (if any). Third, individuals on an approved disability leave were included in the study (*n* = 18) with six of these individuals on long-term disability. It is possible that individuals on disability, particularly those on long-term disability, would not be able to apply the knowledge gained from the course, thus possibly skewing the results. The current study is also limited by a relatively short follow-up period (i.e., 8 weeks). Slow recruitment also resulted in a relatively small sample size, which may impact the generalizability and replicability of results as the sample may not be representative of the larger population [45]. A smaller sample size may have also impacted our ability to detect small effects.

## 6. Future Directions

It is recommended that future research consider using a longer follow-up period to assess longer-term change in accommodation use and symptom management. Beyond improving on methodological issues (e.g., sample size, longer-term assessment, measurement of accommodation requested and received, study after the pandemic), it would be helpful to assess whether improvements to persuasive design of the course (e.g., improving system credibility, personalization) could improve participant engagement and outcome [66]. The inclusion of therapist support also could be examined, similar to internet-delivered cognitive behaviour therapy programs where participants are able to exchange emails or have brief phone calls with therapists [67]. Future studies could then assess if the addition of a therapist helps to facilitate accommodation use and the impacts of accommodations on a variety of outcomes. The inclusion of more information on coping strategies and mental health symptoms may also assist participants in assessing their need for accommodation.

It may also be beneficial to develop a similar course to the WCSC with a focus on employer support. In developing an employer-centered course, it may be helpful to use internationally recognized standards and guidelines for workplace mental health initiatives [68,69]. One such standard is “The National Standard of Canada for Psychological Health and Safety in the Workplace” (the Standard) developed by the MHCC in 2013. The Standard (MHCC; [70]) provides voluntary guidelines to help organizations identify, prevent, and cope with mental health concerns within the workplace. Although the Standard (MHCC; [70]) was developed for organizations specifically, there are components of the Standard that could be addressed within a future course offering. For example, a course offering for employers could include information intended to help support organizations with certain aspects of implementing the guideline such as (a) providing education, awareness, and communication on mental health concerns, (b) identifying preventative and protective measures, and (c) addressing issues related to engagement and change management. This future offering could also contain information on the cost/benefit of implementing accommodations, how to facilitate the accommodation process, the legal aspects of accommodations, and how to talk to employees about their mental health concerns. It could also include a coaching component to help employers navigate complex employment situations. The employer course could also be examined in conjunction with the WCSC to determine the impact of employer support on the accommodation process.

## 7. Conclusions

Overall, this study adds novel information to the literature on workplace accommodations and informs the development and implementation of future courses. The results show some support for the efficacy of the WCSC, suggesting that online education has some potential benefits to employees. Specifically, participants in the WCSC group experienced improved self-efficacy, presenteeism scores, and had more self-reported knowledge of accommodations than those in the WLC. Over time, participants from both groups reported improvements in self-stigmatizing attitudes and anxiety/depression symptoms. In general, the WCSC was successful in many ways and adds to the literature by being the first evidence-based workplace mental health course with a focus on improving accommodation knowledge and use.

## Figures and Tables

**Table 1:** Participant Characteristics by Group at Baseline.

Variable	All Participants (*n* = 77)		Course (*n* = 41)		Wait-List Control (*n* = 36)		Statistical Significance
	*n*	%	*n*	%	*n*	%	
Participant pre-treatment characteristics							
Age							
Mean (*SD*)	43.84 (10.64)	-	44.62 (9.66)	-	43.0 (11.68)	-	*t*(75) = 0.66, *p* = 0.52
Range	20–63	-	26–60	-	20–63	-	
Gender							
Female	60	77.9	31	75.6	29	80.6	χ^2^ (2) = 0.11, *p* = 0.61
Male	16	20.8	9	22.0	7	19.4	
Two-Spirit	1	1.3	1	2.4	0	0	
Ethnicity							
European	59	76.6	30	73.2	29	80.6	χ^2^ (1) = 0.24 *p* = 0.62
Indigenous, Métis, Caribbean, East Asian, Latin American, Not Listed	18	23.4	11	26.8	7	19.4	
Employment characteristics							
Employment status							
Employed full/part time	51	76.3	29	72.5	29	80.6	χ^2^ (1) = 0.31; *p* = 0.58
Disability Leave	18	23.7	11	27.5	7	19.4	
Position							
Executive/senior manager	19	24.7	13	31.7	6	16.7	χ^2^ (3) = 5.72; *p* = 0.13
Professional (engineer, analyst)	20	26.0	8	19.6	12	33.3	
Technical/administrative support	22	28.5	14	34.1	8	22.2	
Sales, service, labourer	16	20.8	6	14.6	10	27.8	
Company Size							
1–99 employees	46	59.7	25	61.0	21	59.7	χ^2^ (2) = 0.09; *p* = 0.96
100–499 employees	16	20.8	8	19.5	8	20.8	
Over 500 employees	15	19.5	8	19.5	7	19.5	
Work functioning (last 28 days)							
Full day absent mean (*SD*)	5.61 (9.92)		6.95 (11.26)		4.08 (8.01)		*t*(75) = 1.29; *p* = 0.19
Partial days absent mean (*SD*)	3.52 (8.27)		4.68 (9.47)		2.19 (6.53)		*t*(75) = 1.36; *p* = 0.18
Performance mean (*SD*)	56.49 (24.64)		53.90 (23.65)		59.44 (25.74)		*t*(75) = 0.98; *p* = 0.33
Mental health characteristics							
Duration of depression concerns							
0–6 months	19	24.7	12	29.3	7	19.4	χ^2^ (2) = 1.11; *p* = 0.58
7–12 months	11	14.3	6	14.6	5	13.9	
>1 year	47	61.0	23	56.12	24	66.7	
Duration of anxiety concerns							
0–6 months	14	18.2	9	22.0	5	18.2	χ^2^ (2) = 1.02; *p* = 0.60
7–12 months	5	6.5	3	7.3	2	5.5	
>1 year	58	75.3	29	70.7	29	75.3	
Pre-treatment symptom scores							
PHQ-9 mean (*SD*)	12.77 (5.53)		12.71 (5.67)		12.83 (5.50)		*t*(75) = 0.09; *p* = 0.92
GAD-7 mean (*SD*)	10.86 (4.54)		10.51 (4.70)		11.25 (4.38)		*t*(75) = 0.71; *p* = 0.48
SPS-6 mean (*SD*)	5.64 (5.21)		5.44 (5.39)		5.86 (5.07)		*t*(75) = 0.35; *p* = 0.72
SIAS-6 mean (*SD*)	8.47 (5.64)		8.19 (5.99)		8.78 (5.29)		*t*(75) = 0.45; *p* = 0.65

Note. PHQ-9 = Patient Health Questionnaire—9 items; GAD-7 = Generalized Anxiety Disorder—7 items; SPS-6 = Social Phobia Scale short form; SIAS-6 = Social Interaction Anxiety Scale Short Form.

**Table 2:** Means, Standard Deviations, and Percent Change for Outcomes by Group.

	Estimated Marginal Means			Percentage Reductions from Pre-Treatment	
	Pre-Treatment	4-Week Follow-up	8-Week Follow-up	to 4-Week Follow-up	to 8-Week Follow-up
AccoKnow ^a^					
Course	4.63 (3.29)	6.53 (2.38)	6.92 (2.12)	−40 [−57, −22]	−47 [−62, −32]
Wait-list Control	3.47 (2.81)	3.66 (2.42)	3.83 (2.51)	−6 [−29, 17]	−11 [−34, 13]
SSMIS (Aware) ^b^					
Course	17.68 (9.85)	15.81 (9.15)	20.45 (11.98)	11 [−7, 29]	−16 [−39, 8]
Wait-list Control	23.83 (10.32)	25.08 (10.49)	26.28 (11.08)	−5 [−20, 9]	−10 [−26, 5]
SSMIS (Agree) ^b^					
Course	12.12 (6.64)	11.89 (6.95)	12.29 (8.01)	2 [−18, 22]	−1 [−24, 21]
Wait-list Control	15.47 (8.72)	12.96 (6.69)	15.01 (7.57)	16 [2, 30]	3 [−13, 19]
SSMIS (Apply) ^b^					
Course	13.02 (6.04)	12.43 (6.92)	12.08 (6.46)	5 [−12, 22]	7 [−9, 23]
Wait-list Control	15.78 (8.51)	14.63 (7.14)	14.95 (6.36)	7 [−7, 22]	5 [−8, 19]
SSMIS (Hurts) ^b^					
Course	14.12 (7.82)	11.45 (7.20)	12.04 (8.75)	19 [3, 35]	15 [−5, 34]
Wait-list Control	16.78 (9.03)	14.49 (8.58)	14.39 (7.77)	14 [−3, 30]	14 [−1, 30]
WRS ^b^					
Course	40.10 (12.66)	39.86 (11.40)	38.82 (10.82)	1 [−9, 10]	3 [−5, 11]
Wait-list Control	39.75 (11.11)	39.63 (9.99)	40.19 (9.88)	0 [−8, 8]	−1 [−9, 7]
WHOHPQ (PD) ^b^					
Course	3.80 (7.02)	3.51 (6.96)	2.98 (7.18)	8 [−55, 71]	22 [−44, 87]
Wait-list Control	2.19 (6.53)	4.49 (9.57)	2.71 (8.13)	−104 [−249, 40]	−23 [−148, 101]
WHOHPQ (Pre) ^a^					
Course	53.90 (23.65)	55.20 (26.37)	62.24 (20.46)	−2 [−18, 13]	−15 [−29, −2]
Wait-list Control	59.44 (25.74)	56.20 (26.79)	55.08 (27.28)	5 [−9, 20]	7 [−7, 22]
NGSE ^a^					
Course	3.29 (0.74)	3.51 (0.70)	3.73 (0.66)	−8 [−15, −1]	−13 [−20, −6]
Wait-list Control	3.43 (0.73)	3.49 (0.69)	3.49 (0.68)	−2 [−8, 4]	−2 [−8, 5]
DISC (Comfort Full) ^a^					
Course	2.59 (0.89)	2.71 (0.75)	2.80 (0.79)	−5 [−14, 4]	−8 [−18, 2]
Wait-list Control	2.36 (0.87)	2.54 (0.79)	2.43 (0.91)	−7 [−18, 4]	−3 [−15, 10]
DISC (Comfort Partial) ^a^					
Course	2.29 (0.75)	2.54 (072)	2.53 (0.78)	−11 [−22, 0]	−10 [−21, 1]
Wait-list Control	2.42 (0.87)	2.57 (0.71)	2.66 (0.85)	−6 [−16, 3]	−10 [−22, 1]
DISC (Rate) ^a^					
Course	2.05 (0.71)	2.16 (0.66)	2.35 (0.54)	−5 [−16, 5]	−15 [−24, −6]
Wait-list Control	2.11 (0.57)	2.10 (0.67)	2.17 (0.64)	0 [−10, 11]	−3 [−13, 7]
PHQ-9 ^b^					
Course	12.71 (5.67)	9.31 (5.98)	9.08 (7.26)	27 [12, 42]	29 [11, 46]
Wait-list Control	12.83 (5.46)	9.44 (4.86)	9.70 (5.03)	26 [14, 39]	24 [12, 37]
GAD-7 ^b^					
Course	10.51 (4.70)	7.58 (4.29)	7.54 (5.97)	28 [14, 41]	28 [10, 47]
Wait-list Control	11.25 (4.38)	8.40 (5.12)	9.40 (5.00)	25 [10, 40]	16 [2, 31]
SPS-6 ^b^					
Course	5.44 (5.39)	4.83 (6.05)	4.92 (6.01)	11 [−22, 45]	10 [−24, 43]
Wait-list Control	5.86 (5.07)	6.11 (5.91)	5.94 (5.20)	−4 [−37, 29]	−1 [−32, 29]
SIAS-6 ^b^					
Course	8.20 (5.99)	6.96 (5.97)	7.23 (6.28)	15 [−7, 37]	11 [−12, 35]
Wait-list Control	8.78 (5.29)	8.36 (5.96)	8.55 (5.67)	5 [−18, 27]	3 [−18, 24]

Note. Standard deviations are shown in rounded parentheses for estimated means; 95% confidence intervals are shown in square parentheses for percentage changes. AccoKnow = Accommodation Knowledge; SSMIS = Self-Stigma of Mental Illness Scale Short Form (subscales: Awareness/Agreement/Application/Hurts self); WRS = Workers’ Relation Scale; WHO HPQ = World Health Organization Health and Work Performance Questionnaire (PD = Partial day absent; Pre = Presenteeism); NGSE = New General Self-Efficacy scale; DISC = Disclosure (Comfort disclosing full; Comfort disclosing partial; Disclosure rate); PHQ-9 = Patient Health Questionnaire 9-item; GAD-7 = Generalized Anxiety Disorder 7-item; SPS-6 = Social Phobia Scale Short Form; SIAS-6 = Social Interaction Anxiety Scale Short Form. ^a^ Statistics were calculated on additive or subtractive changes. ^b^ Statistics were calculated on proportional percent change.

**Table 3:** Within-Group and Between-Group Effect Sizes (Cohen’s d) at 4-week and 8-week follow-up.

	Within-Group Effect Sizes from Pre-Treatment		Between-Group Effect Size	
	to 4-Week Follow-up	to 8-Week Follow-up	at 4-Week Follow-up	at 8-Week Follow-up
AccoKnow				
Course	−0.65 [−1.09, −0.20]	−0.81 [−1.26, −0.36]	1.17 [0.69, 1.66]	1.31 [0.82, 1.80]
Wait-list Control	−0.07 [−0.53, 0.39]	−0.13 [−0.60, 0.33]		
SSMIS (Aware)				
Course	0.19 [−0.24, 0.63]	−0.25 [−0.68, 0.19]	−0.93 [−1.40, −0.46]	−0.49 [−0.95, −0.04]
Wait-list Control	−0.12 [−0.58, 0.35]	−0.22 [−0.69, 0.24]		
SSMIS (Agree)				
Course	0.03 [−0.40, 0.47]	−0.02 [−0.46, 0.41]	−0.15 [−0.60, 0.29]	−0.34 [−0.79, 0.11]
Wait-list Control	0.32 [−0.15, 0.78]	0.06 [−0.41, 0.52]		
SSMIS (Apply)				
Course	0.09 [−0.34, 0.52]	0.15 [−0.28, 0.58]	−0.31 [−0.76, 0.14]	−0.44 [−0.89, 0.01]
Wait-list Control	0.14 [−0.32, 0.61]	0.11 [−0.35, 0.57]		
SSMIS (Hurts)				
Course	0.35 [−0.09, 0.78]	0.25 [−0.19, 0.68]	−0.38 [−0.83, 0.07]	−0.28 [−0.73, 0.17]
Wait-list Control	0.25 [−0.21, 0.72]	0.28 [−0.19, 0.74]		
WRS				
Course	0.02 [−0.41, 0.45]	0.11 [−0.33, 0.54]	0.02 [−0.43, 0.47]	−0.13 [−0.58, 0.32]
Wait-list Control	0.01 [−0.45, 0.47]	−0.04 [−0.50, 0.42]		
WHOHPQ (PD)				
Course	0.04 [−0.39, 0.47]	0.11 [−0.32, 0.55]	−0.12 [−0.56, 0.33]	0.04 [−0.41, 0.48]
Wait-list Control	−0.27 [−0.74, 0.19]	−0.07 [−0.53, 0.39]		
WHOHPQ (Pre)				
Course	−0.05 [−0.48, 0.38]	−0.37 [−0.81, 0.07]	−0.04 [−0.48, 0.41]	0.29 [−0.16, 0.74]
Wait-list Control	0.12 [−0.34, 0.58]	0.16 [−0.30, 0.62]		
NGSE				
Course	−0.29 [−0.73, 0.14]	−0.62 [−1.06, −0.17]	0.02 [−0.42, 0.47]	0.35 [−0.10, 0.80]
Wait-list Control	−0.08 [−0.54, 0.38]	−0.09 [−0.55, 0.37]		
DISC (Comfort Full)				
Course	−0.15 [−0.59, 0.28]	−0.25 [−0.68, 0.19]	0.23 [−0.22, 0.68]	0.43 [−0.02, 0.88]
Wait-list Control	−0.21 [−0.67, 0.26]	−0.07 [−0.54, 0.39]		
DISC (Comfort Partial)				
Course	−0.33 [−0.77, 0.11]	−0.30 [−0.74, 0.13]	−0.04 [−0.49, 0.40]	−0.16 [−0.61, 0.29]
Wait-list Control	−0.19 [0.66, 0.27]	−0.28 [−0.75, 0.18]		
DISC (Rate)				
Course	−0.16 [−0.59, 0.28]	−0.47 [−0.91, −0.04]	0.08 [−0.37, 0.53]	0.30 [−0.15, 0.75]
Wait-list Control	0.01 [−0.45, 0.47]	−0.10 [−0.56, 0.36]		
PHQ-9				
Course	0.57 [0.13, 1.01]	0.55 [0.11, 0.99]	−0.02 [−0.47, 0.42]	−0.10 [−0.54, 0.35]
Wait-list Control	0.64 [0.17, 1.12]	0.58 [0.11, 1.06]		
GAD-7				
Course	0.64 [0.20, 1.08]	0.54 [0.10, 0.98]	−0.17 [−0.62, 0.28]	−0.33 [−0.78, 0.12]
Wait-list Control	0.59 [0.11, 1.06]	0.38 [−0.08, 0.85]		
SPS-6				
Course	0.10 [−0.33, 0.54]	0.09 [−0.34, 0.52]	−0.21 [−0.66, 0.24]	−0.18 [−0.63, 0.27]
Wait-list Control	−0.04 [−0.51, 0.42]	−0.01 [−0.48, 0.45]		
SIAS-6				
Course	0.20 [−0.23, 0.64]	0.15 [−0.28, 0.58]	−0.23 [−0.68, 0.22]	−0.21 [−0.66, 0.24]
Wait-list Control	0.07 [−0.39, 0.53]	0.04 [−0.42, 0.50]		

Note. 95% confidence intervals are shown in square parentheses. AccoKnow = Accommodation Knowledge; SSMIS = Self-Stigma of Mental Illness Scale Short Form (subscales: Awareness/Agreement/Application/Hurts self); WRS = Workers’ Relation Scale; WHO HPQ = World Health Organization Health and Work Performance Questionnaire (PD = Partial day absent; Pre = Presenteeism); NGSE = New General Self-Efficacy scale; DISC = Disclosure (Comfort disclosing full; Comfort disclosing partial; Disclosure rate); PHQ-9 = Patient Health Questionnaire 9-item; GAD-7 = Generalized Anxiety Disorder 7-item; SPS-6 = Social Phobia Scale Short Form; SIAS-6 = Social Interaction Anxiety Scale Short Form.

## Data Availability

The data presented in this study are available upon request from the corresponding author.
